# Biological effects of exosomes derived from 2D and 3D culture adipose stem cells on JEC Cell proliferation and migration

**DOI:** 10.3389/fbioe.2025.1541150

**Published:** 2025-07-10

**Authors:** Yifen Li, Gangwei Wang, Yi Zhang, Tao Xu, Xuefeng Jiang

**Affiliations:** ^1^ Gynecology Department of the Third Affiliated Hospital of Sun Yat sen University, Guangzhou, Guangdong, China; ^2^ Emergency Department of the Third Affiliated Hospital of Sun Yat sen University, Guangzhou, Guangdong, China; ^3^ Precision Medicine and Healthcare Research Center, Tsinghua-Berkeley Shenzhen Institute (TBSI), Tsinghua University, Shenzhen, Guangdong, China; ^4^ Obstetrics and Gynecology Department of the First Affiliated Hospital of Jinan University, Guangzhou, Guangdong, China

**Keywords:** adipose-derived stem cells (ADSCs), exosomes, endometrial cancer, 2D culture, 3D culture, proliferation, invasion, migration

## Abstract

**Objective:**

To explore the biological impacts of exosomes derived from adipose stem cells cultured under two-dimensional (2D) and three-dimensional (3D) conditions on endometrial adenocarcinoma cells (JEC cells).

**Materials and methods:**

Exosomes were isolated and extracted from adipose stem cells grown in 2D and 3D cultures. JEC cells were allocated into three categories: a control group, a 2D group, and a 3D group. Cultivation of JEC cells occurred with distinct media (supplemented with or without 2D/3D adipose-derived stem cell exosomes) per group. Endometrial cancer cell viability was evaluated by a Cell Counting Kit-8 (CCK-8) assay. Scratch assays gauged the migratory behavior of endometrial adenocarcinoma cells. A Transwell assay quantified cellular invasiveness.

**Results:**

The 3D group exhibited amplified cell proliferation (p < 0.05). Higher wound closure rates at 72 h were observed in the 3D group (p < 0.05). The Transwell assay demonstrated a substantial rise in the number of JEC cells traversing the Transwell chamber within the 3D group at 48 h (p < 0.05).

**Conclusion:**

Exosomes obtained from 2D and 3D adipose stem cells significantly bolstered cell proliferation, invasion, and migration. Exosomes sourced from 3D adipose-derived stem cells displayed greater efficacy than their 2D counterparts.

## 1 Introduction

Endometrial cancer (EC), recognized as one of the three primary gynecological malignancies, is notably influenced by escalated estrogen levels, metabolic abnormalities such as obesity and diabetes, and a genetic susceptibility to the disease (Chinese Society of Gynecological Oncology, 2021). Human adipose tissue not only serves as an energy reservoir but also acts as an endocrine organ, engaging in metabolism, immune responses, and the synthesis of bioactive substances. It is implicated in cellular growth and differentiation, angiogenesis, cell apoptosis, and carcinogenesis. Abundant estrogen receptors in adipose tissue impact body fat distribution (Zhao et al., 2005). At specific concentrations, estrogen markedly enhances the proliferation and directed differentiation of adipose-derived stem cells (Ren et al., 2013). The literature reviews revealed that obesity, coupled with altered adipokine levels—including adiponectin, leptin, and resistin—is intimately tied to the risk and prognosis of endometrial cancer (Michalczyk et al., 2021; Garikapati et al., 2019). Consequently, there might exist a connection between adipose tissue and the onset and progression of endometrial cancer.

ADSCs (adipose-derived stem cells), owing to their abundant availability, ease of procurement, rapid *in vitro* proliferation capabilities, and high stem cell ratio, are emerging as a powerful tool in cell therapy and regenerative medicine. These cells secrete exosomes that inherit diverse functions, including cytokine secretion, maintenance of homeostasis, regulation of immune responses, and promotion of neurogenesis and angiogenesis. Exosomes not only facilitate biomolecule transfer between different cell types but also mediate intercellular communication, impacting both normal physiological metabolism and disease regulation within the human body. In recent years, exosomes have emerged as promising biomarkers, cell-free therapeutic agents, and drug delivery carriers. Despite their significant clinical potential, efficient production of exosomes in sufficient quantities remains a challenge (Hong et al., 2019). Utilizing 3D culture methods, such as scaffolds and bioreactors, allows for the expansion of large numbers of cells and, consequently, the production of exosomes. The application of 3D culture systems has been demonstrated to increase the yield of exosomes, reducing labor, time, and costs, while offering superior therapeutic outcomes compared to 2D exosomes. Moreover, 3D cell spheroids exhibit enhanced clinical and biological practicality due to their superior regeneration potential (Costa et al., 2017).

In the study of endometrial cancer pathogenesis, numerous investigations have revealed that exosomes play a pivotal role in the disease’s pathogenesis, directly influencing the biological functions of tumor cells and orchestrating communication between tumor cells and the tumor microenvironment. For example, exosomes derived from platelets have been shown to enhance the proliferation and migration of endometrial stromal cells and the endometrial cancer cell line, HEC-1A (Miller et al., 2022). Exosomes secreted by human umbilical cord mesenchymal stem cells (hUCMSCs) carry miRNA-503-3p, which inhibits the progression of endometrial cancer by downregulating the mesoderm-specific transcript (MEST) (Pan et al., 2022). CD8^+^ T cell-derived miR-765 inhibits the progression of endometrial cancer via the ERβ/miR-765/PLP2/Notch axis (Zhou WJ. et al., 2021). Moreover, exosomes from the serum of patients with polycystic ovary syndrome (PCOS) augment the invasive and migratory capacities of endometrial cancer cells, facilitating their migration and invasion (Che et al., 2020). Collectively, these investigations underscore the intricate and multifaceted functions of exosomes in the etiology and advancement of endometrial cancer. Despite the promising clinical prospects of exosomes, research on the effects of ADSC exosomes on endometrial cancer remains sparse, especially concerning the comparative efficacy of 2D versus 3D exosome derivations. Estrogen, obesity, and diabetes constitute well-established risk factors for the development and progression of endometrial and breast cancers. Emerging evidence indicates that ADSC-derived exosomes (ADSCs-Exos) from diabetic patients may remodel the tumor microenvironment, potentially promoting the growth of estrogen receptor (ER)-positive breast cancer (Jafari et al., 2021). Both obesity and diabetes can significantly alter ADSC functionality, enhancing adipogenic differentiation capacity and triglyceride production (Meng et al., 2019a; Tang, 2017; Li et al., 2018). Notably, ADSCs-Exos from obese and diabetic patients exhibit distinct genetic profiles, with their miRNA cargo preferentially involved in pro-inflammatory and lipogenic pathways compared to healthy controls, thereby exacerbating metabolic syndrome-related complications (Meng et al., 2019b).

This study endeavors to evaluate the influence of 2D and 3D cultured ADSC exosomes on the proliferation, invasion, and migration of endometrial adenocarcinoma cells, given the established link between adipose tissue and endometrial cancer risk. The results from this investigation will enhance our comprehension of the role of ADSC exosomes in endometrial cancer progression and could provide novel therapeutic targets for endometrial cancer intervention strategies.

## 2 Materials and methods

### 2.1 Collection of human adipose tissue and extraction of ADSCs

Adipose tissue specimens were procured from the subcutaneous adipose tissue of nine non-endometrial cancer patients undergoing abdominal surgery at the Gynecology Department of the Third Affiliated Hospital of Sun Yat-sen University in Guangzhou. Patients’ ages ranged between 40 and 60 years, with no history of infection or malignancies, and no presence of other endocrine or systemic metabolic disorders. The study was approved by the hospital’s Medical Ethics Committee, and informed consent forms were signed by the patients. Adipose tissue was collected under sterile conditions, with a volume ranging from 100 to 500 mL, into sterile containers and stored at 2°C–8°C. It was ensured that the entire process from collection to processing was completed within 4 hours. Samples were retained for inspection during preparation. Adipose tissue was transferred into 50 mL centrifuge tubes for temporary storage, with 20 mL of tissue per tube, followed by rinsing with 30 mL of PBS. Centrifugation was conducted at room temperature at 700 rpm for 5 minutes, after which the lower mixed solution was discarded, and the upper layer of adipose tissue lumps was retained. Collagenase Type I was diluted to a concentration of 1 μg/mL with DMEM, and the enzyme solution was added to the adipose tissue lumps in a 1:1 ratio. After thorough mixing, the tissue was incubated at 37°C on a shaker at 170 rpm for 45 min until the tissue had liquefied. Centrifugation at 20°C at 1,300 rpm for 5 min followed, with the fat and supernatant being discarded.

The cellular precipitate was rinsed with 10 mL of PBS one to two times. Centrifugation at 20°C at 1,300 rpm for 5 minutes ensued, with the supernatant removed. Cells were resuspended in 1 mL of MSCM. Then, 10 mL of MSCM was added to a 100 mm culture dish, and the cells were inoculated at 100 w/dish into a 100 mm culture dish, mixed well, and marked with identifiers for the cells, passage number, and date. Cells were cultured in an incubator set at 37°C with 5% CO2. Subsequent experiments were performed using third-generation cells. All ADSCs were isolated from the same donor through enzymatic digestion and subsequently divided into two groups: one group underwent conventional 2D culture, while the other was seeded into a combined platform of MSC microfiber culture and ultracentrifugation purification for expansion.

### 2.2 ADSCs-Exos extraction

ADSCs from each donor were split into two parallel cultures. For the extraction of 3D ADSCs-Exos, we utilized a combined platform of MSC microfiber culture and ultracentrifugation purification, encapsulating a large volume of MSC solution (approximately 3 × 10^8^ total cells) in 1-m long hollow hydrogel microfibers through co-axial bioprinting technology (Chen et al., 2021). In this 3D core-shell microfiber environment, the levels of stemness markers (Oct4, Nanog, Sox2) expressed by MSCs were higher than in 2D culture, while maintaining their differentiation potential. Compared with traditional 2D culture, this platform enriched particles by approximately 1,009 times while preserving their angiogenic properties. the third-generation 2D adipose stem cells were conventionally cultured for 48 h. The cell supernatant from 2D and 3D ADSCs were colected seprately,then we centrifuged them at 1,000 g for 5 min to remove cellular precipitates, and the exosomes in the cell lysate were extracted according to the instructions of the exosome extraction kit. The concentration of exosomes was adjusted to 500 μg/mL using 0.5% FBS DMEM medium.

### 2.3 Cell culture

Cells were maintained in a humidified incubator at 37°C. Human endometrial adenocarcinoma cells (JEC) were cultured in RPMI-1640 medium supplemented with 10% fetal bovine serum. Adipose-derived stem cell exosomes (ADSCs-Exos), sourced from Shenzhen Huaqing Zhimei Bio Co., Ltd., were used for 2D and 3D conditions. Morphology of the ADSCs-Exos was characterized using transmission electron microscopy (TEM). Nanoparticle tracking analysis (NTA) was utilized to determine the size and distribution of both 2D and 3D ADSCs-Exos. Western blot was conducted to ascertain the expression levels of exosomal protein markers including calnexin, CD63, CD81, and TSG101.

### 2.4 Cell proliferation

For assessing cell proliferation, JEC cells were segregated into a 2D group, a 3D group, and a control group, with each group seeded at a density of 1 × 10^4^ cells per well in 96-well plates. Cells in the 2D and 3D groups were cultured in RPMI-1640 medium containing ADSCs-Exos at a concentration of 5 × 10^7^ particles/mL, whereas the control group received an equivalent volume of RPMI 1640 without exosomes. After 24 h of incubation, cell viability was quantified through the CCK8 colorimetric method. Ten microliters of the CCK8 reagent were added to each well and incubated for 2 h. Absorbance was then measured at 490 nm using a microplate reader. Growth curves were generated based on the optical density (OD) values obtained. All experiments were executed in triplicate.

### 2.5 *In vitro* wound healing

JEC cells were plated until they reached a confluent monolayer (approximately 90%–100%). A uniform scratch was produced across the wells. Post-scratch, the cells were rinsed with phosphate-buffered saline (PBS). Subsequently, 2D/3D ADSC EXOs (with an exosome concentration of 2 × 10^9^ particles/mL) were applied to each group separately. The control group received an equivalent volume of serum-free medium. Images were recorded every 12 h, and each experiment was replicated three times.

### 2.6 Western blotting

We separately extracted proteins from the 2D and 3D ADSC exosomes. The protein samples were subjected to separation through 10% Sodium Dodecyl Sulfate Polyacrylamide Gel Electrophoresis (SDS-PAGE). The gel was immersed in electrophoresis buffer, and the protein samples were loaded. An electric current was applied, causing the proteins to migrate from the cathode to the anode of the gel. Subsequently, the proteins were electro-transferred onto a polyvinylidene difluoride (PVDF) membrane. The membrane was then placed in a container, covered with blocking buffer, and the antibodies were diluted at their recommended concentrations in the same buffer. The primary antibody incubation took place overnight at 4°C, followed by a 4-h incubation with the appropriate secondary antibody. Bands were visualized using an enhanced chemiluminescence (ECL) kit.

### 2.7 Cell invasion assay

Transwell chambers (pore size: 8 μm) were utilized to evaluate cell invasiveness. JEC cells were divided into experimental and control groups. The upper chambers were coated with Matrigel. For the experimental group, the upper chamber was filled with 500 μL of RPMI 1640 medium containing a concentration of 5 × 10^7 particles/mL of 2D/3D ADSCs-Exos. The control group received an equivalent volume of serum-free medium. The lower chamber was supplied with 10% fetal bovine serum (FBS). After 48 h of incubation, the chambers were retrieved and the fluid removed. The invading cells were fixed with 20% methanol for 30 min and stained with a 0.5% crystal violet solution for 10 min. Images were captured using a fluorescent inverted microscope for subsequent analysis.

### 2.8 Exosome uptake analysis

The isolated 2D and 3D ADSCs-Exos were labeled with PKH67 according to the manufacturer’s protocol. The fluorescently-labeled exosomes were co-incubated with JEC cells for 24 h at 37°C. Counter-staining with DAPI was performed, and observation took place under a laser confocal microscope. The appearance of green fluorescence indicated successful uptake of the exosomes by the cells.

### 2.9 RNA-seq analysis

Three samples from each group were subjected to RNA-seq analysis. Total RNA was extracted using the exoRNeasy Serum/Plasma Maxi Kit. Total RNA was digested with RNase R. Strand-specific RNA-seq libraries were prepared using the VAHTS Total RNA-seq (H/M/R) Library Prep Kit for Illumina. The digested RNA samples were fragmented and then subjected to first- and second-strand cDNA synthesis using random hexamer primers. During the second-strand synthesis reaction, dTTP was replaced with dUTP. After cDNA synthesis, the double-stranded products underwent end-repair, the addition of a single ‘a' base, and adapter ligation to the cDNA products. cDNA fragments were captured using magnetic particles and treated with uracil DNA glycosylase to remove the second-strand cDNA. The purified first-strand cDNA was PCR-amplified, and the libraries were analyzed using a Bioanalyzer 4200. Sequencing of the cDNA was then performed on the Illumina HiSeq X Ten (X10) system. Identification of Differentially Expressed miRNAs Differential gene expression in the RNA-seq data was calculated using DEseq2/edgeR software. To infer potential functions of the differentially expressed miRNAs, the miRNA gene symbols were submitted to the web-based tool DAVID for Reactome pathway enrichment analysis.

### 2.10 Statistical analysis

The statistical analysis was conducted using GraphPad Prism 8 (GraphPad Software, CA, United States). Each experiment was replicated independently three times. The data are displayed as mean ± standard deviation (SD). Comparisons across multiple groups were assessed employing one-way ANOVA and repeated-measures ANOVA. A p-value less than 0.05 was considered statistically significant.

## 3 Results

### 3.1 Characterization of 2D and 3D ADSCs-Exos

**FIGURE 1:**
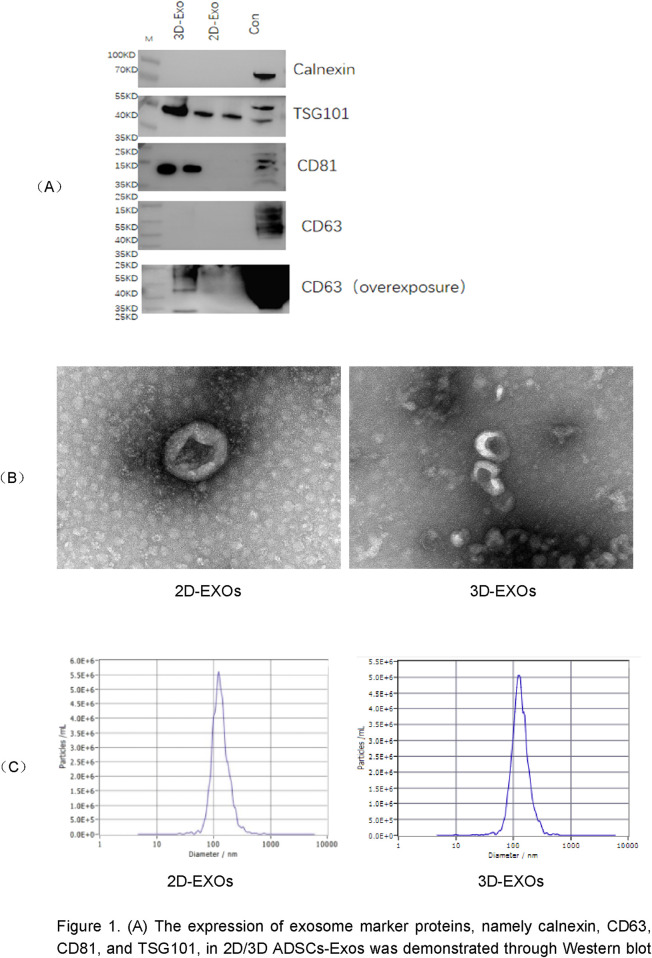
**(A)** The expression of exosome marker proteins, namely calnexin, CD63, CD81, and TSG101, in 2D/3D ADSCs-Exos was demonstrated through Western blot analysis. **(B)** Transmission electron microscopy (TEM) images revealed the characteristic morphology of 2D/3D ADSCs-Exos, featuring spherical vesicles with hypodense centers and hyperdense lipid bilayers. **(C)** Particle tracking analysis (NTA) was employed to determine the size and concentration of exosomes within the 2D/3D ADSCs-Exos samples.

By Western blotting, 2D/3D ADSCs-Exos showed the expression of exosomal marker proteins Calnexin, CD63, CD81, and TSG101 (Figure 1A). Transmission electron microscopy (TEM) images demonstrated the characteristic exosome morphology of 2D/3D ADSCs-Exos, including spherical vesicles with hypodense centers and hyperdense lipid bilayers (Figure 1B). The concentrations of 2D- and 3D-derived exosomes were quantified using Nanoparticle Tracking Analysis (NTA) (Figure 1C). The results demonstrated that 3D-cultured exosomes from the same donor exhibited a concentration of 3.1 × 10^11^ particles/mL, which was 2.8-fold higher than that of 2D-cultured exosomes (1.1 × 10^11^ particles/mL) (Table 1), confirming that the 3D system significantly enhanced exosome yield. The average size of 3D exosomes was approximately 139.7 nm with a concentration of 3.1 × 10^11^ particles per ml (Figure 1C; Table 1).

**TABLE 1:** Exosome size and concentration of 2D/3D ADSCs-Exos.

Sample	Average grain diameter (nm)	Concentration (Particles/mL)
2D	139.5	1.1 × 10^11^
3D	139.7	3.1 × 10^11^

### 3.2 Absorption of 2D and 3D ADSCs-Exos by JEC cells

**FIGURE 2:**
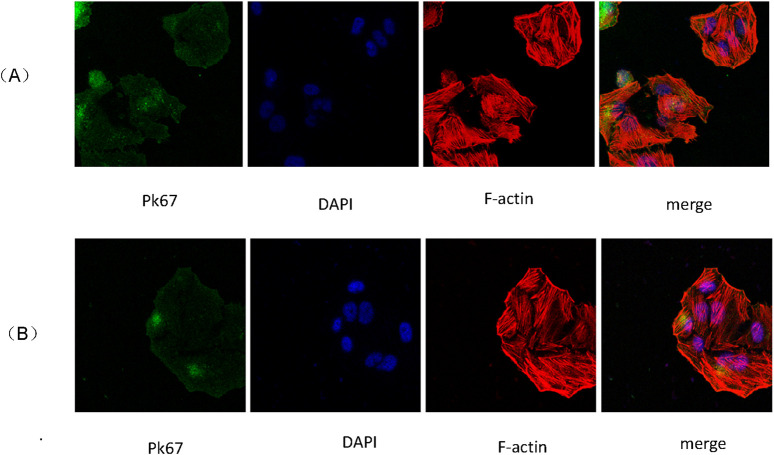
**(A)** Uptake of 2D ADSCs-Exos by JEC cells; **(B)** Uptake of 3D ADSCs-Exos by JEC cells.

Scanning electron microscopy revealed that JEC cells internalized 2D and 3D ADSCs-Exos treated with 1 × 10^12^ particles/mL for periods of 24–48 h (Figures 2A,B). This observation underscored the ability of 2D and 3D ADSCs-Exos to engage with endometrial cells.

### 3.3 Functional changes in endometrial cells following 2D and 3D ADSCs-Exos treatment

**FIGURE 3:**
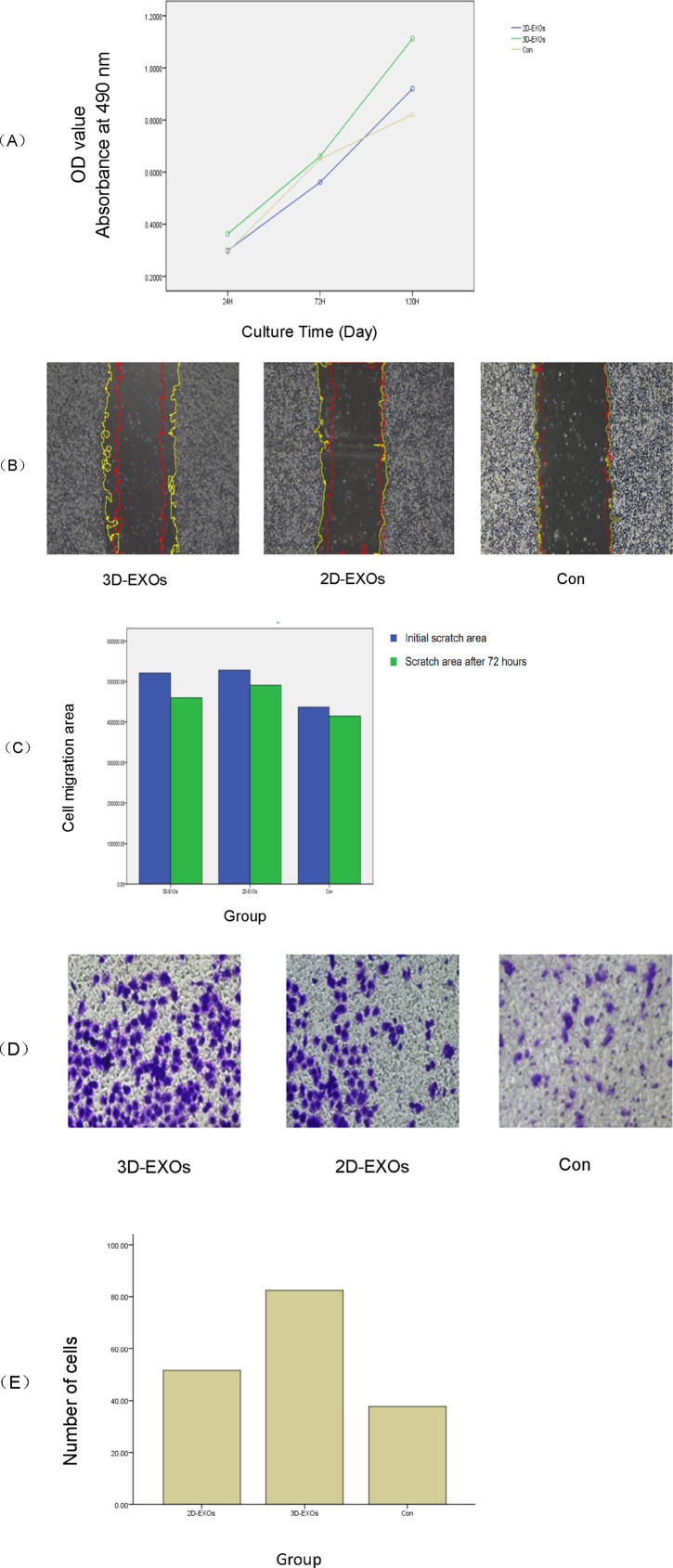
**(A)** The CCK8 assay demonstrated that at 72 h post-treatment, the optical density (OD) values of JEC cells exposed to 3D ADSCs-Exos were significantly higher compared to those treated with 2D ADSCs-Exos and the control group (*p* < 0.05). Notably, cell proliferation in the 3D group markedly increased by the fifth day (*p* < 0.05). **(B)** By 72 h, both the 2D and 3D groups showed enhanced cell migration. There were significant differences between the treatment groups and the control at 72 h, with the 3D group exhibiting the highest healing rate *(p* < 0.05). The yellow line represents the initial contour, and the red line represents the cell migration contour after 72 h. **(C)** Bar graph showing the mean initial and 72-h post-scratch wound areas of each group. **(D)** The cell invasion assay revealed that at 48 h, JEC cells treated with 3D ADSCs-Exos possessed the strongest invasive capacity among all groups (*p* < 0.05). **(E)** Bar graph of the number of invasive cells in each group.

Functionally, alterations in endometrial cells subsequent to 2D and 3D ADSCs-Exos treatments were evident. The CCK-8 assay demonstrated an enhanced optical density (OD) value in JEC cells exposed to 5 × 10^7^ particles/mL 3D ADSCs-Exos at 72 h, outperforming cells subjected to 5 × 10^7^ particles/mL 2D ADSCs-Exos and serum-free media. Moreover, a significant increase in cell proliferation was noted in the 3D group on the fifth day (Inter-group differences: F = 3.331 *p* = 0.026), as depicted in Figure 3A. Specific OD values at each time point for each group are detailed in Table 2.

**TABLE 2:** The OD values in each group.

Group	24H (OD490nm)	72H (OD490nm)	120H (OD490nm)
2D	0.30 ± 0.011	0.561 ± 0.057	0.919 ± 0.147
3D	0.363 ± 0.061	0.661 ± 0.161	1.112 ± 0.105
Control	0.295 ± 0.049	0.65 ± 0.048	0.821 ± 0.166

F = 3.331, *p* = 0.026, n = 3 independent experiments.

A scratch assay was employed to gauge the wound healing potential within each group. At 72 h post-treatment, JEC cells exposed to 2 × 10^9^ particles/mL 2D and 3D ADSCs-Exos exhibited increased migration compared to cells treated with serum-free media. Statistically significant differences were observed between the treatment groups and the control at 72 h, with the 3D group boasting the highest wound healing rate (F = 59.575 *p* = 0.000), as shown in Figures 3B,C. The migration area in each group is provided in Table 3.

**TABLE 3:** The migration area in each group.

Group	Initial scratch area (pixels)	Scratch area after 72 h (pixels)
2D	5.28E5 ± 3.87E4	4.91E5 ± 4.02E4
3D	5.21E5 ± 5.59E4	4.59E5 ± 2.45E4
Control	4.37E5 ± 1.69E4	4.15E5 ± 1.13E4

F = 59.575, *p* = 0.000, n = 3 independent experiments.

(3D vs. 2D: *p* = 0.000, 3D vs. Con: *p* = 0.013).

As per the cell invasion assay, JEC cells treated with 5 × 10^7^ particles/mL 3D ADSCs-Exos showcased the most robust invasiveness at 48 h, with statistical significance (F = 8.085 *p* = 0.006), as illustrated in Figures 3D,E. The mean number of cells traversing the transwell chamber for each group is provided in Table 4.

**TABLE 4:** The number of invading cells in each group after 48 h.

Group	The number of invading cells after 48 h
2D	51.6 ± 8.02
3D	82.4 ± 27.042
Control	37.8 ± 13.10

F = 8.085, p = 0.006, n = 3 independent experiments.

(3D vs. 2D: p = 0.046, 3D vs. Con: p = 0.005).

### 3.4 Results of differentially expressed genes and reactome enrichment pathways

**FIGURE 4:**
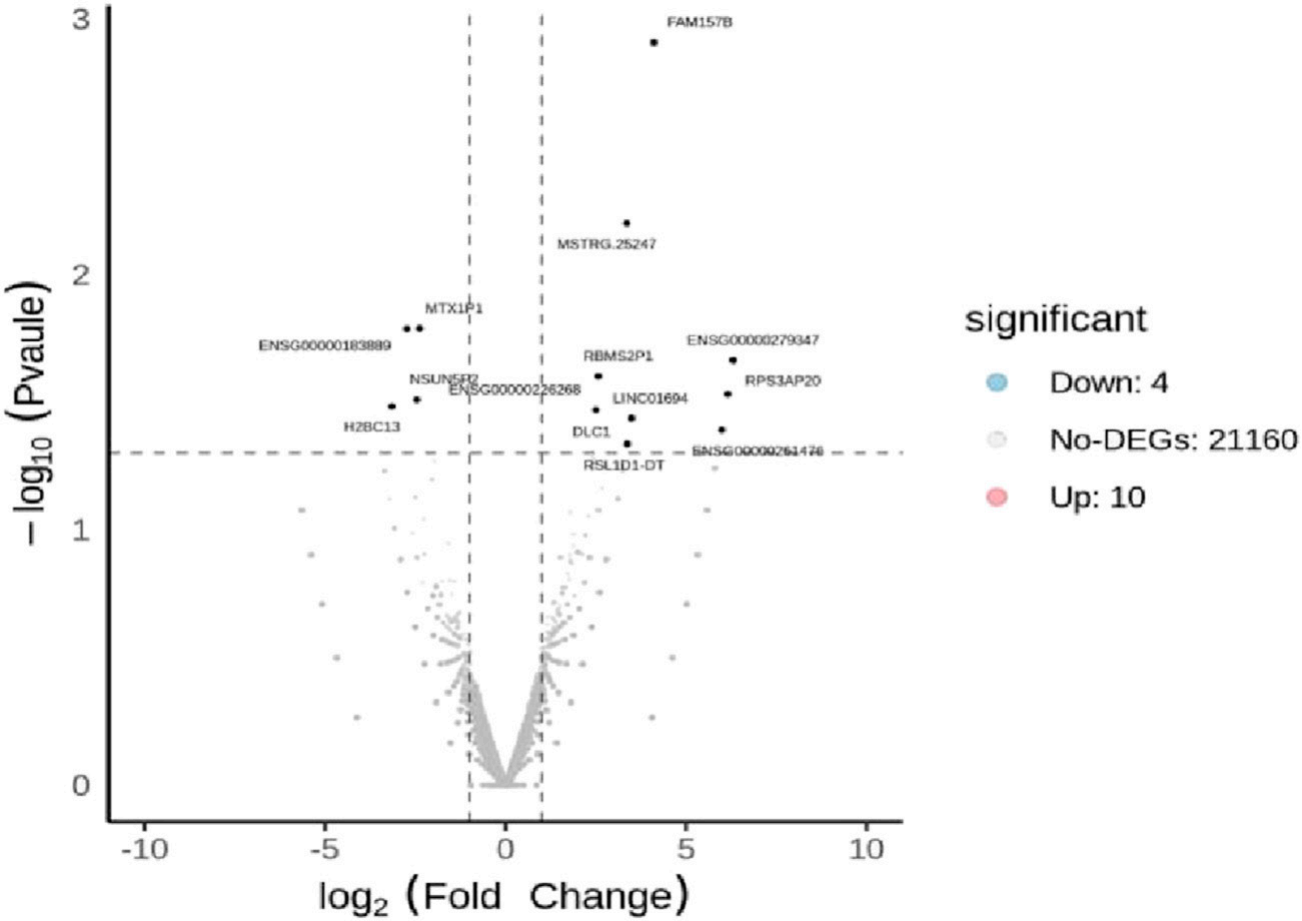
Volcano plot of differentially expressed genes.

**FIGURE 5:**
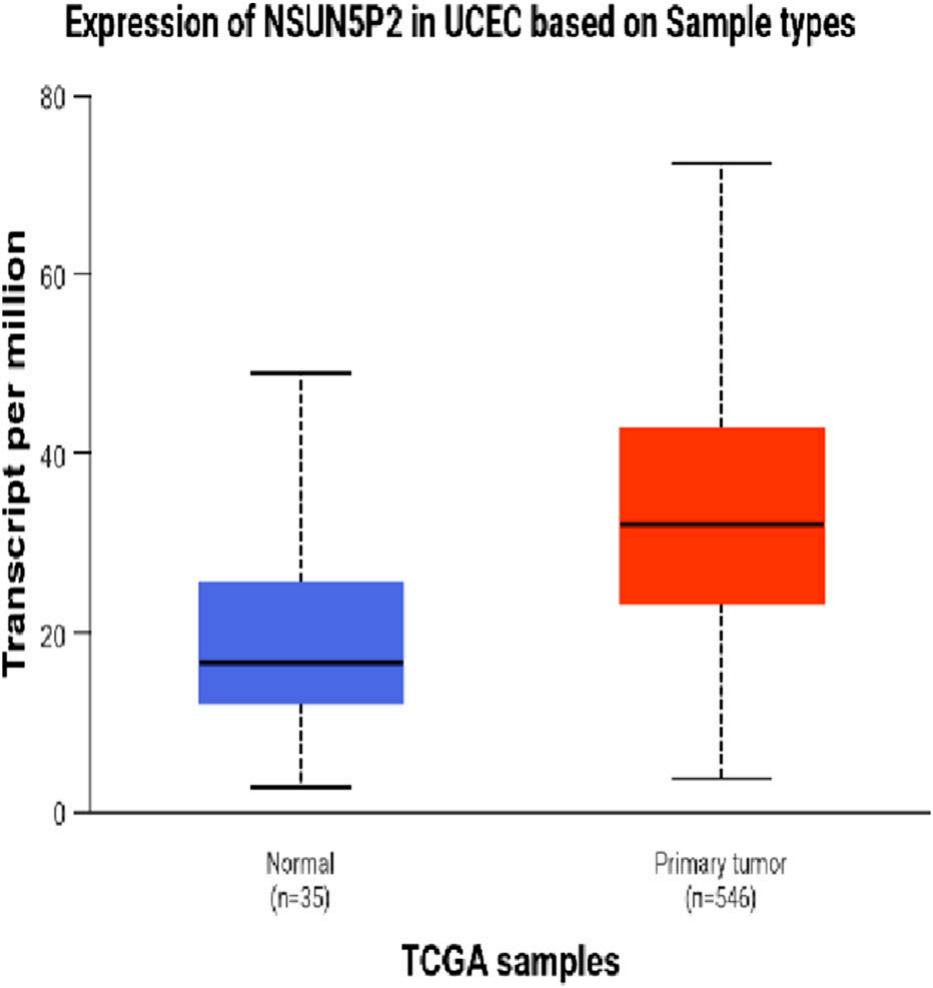
Expression of NSUN5P2 in UCEC.

**FIGURE 6:**
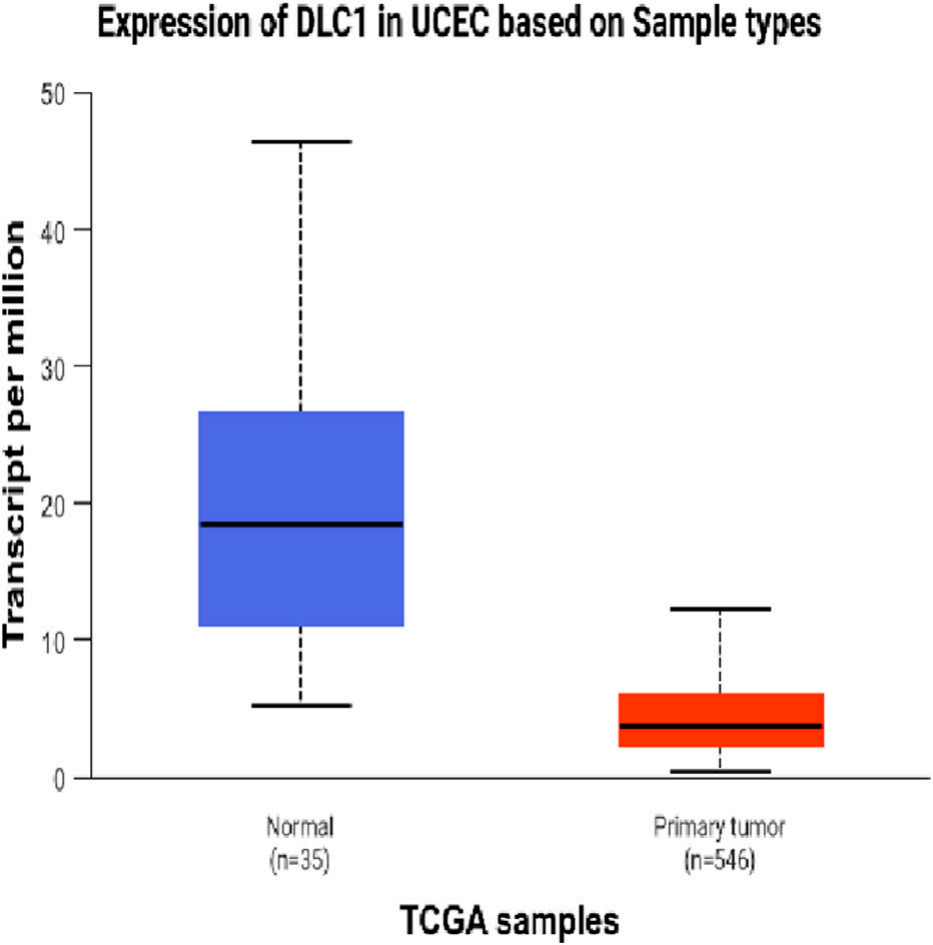
Expression of DLC1 in UCEC.

**FIGURE 7:**
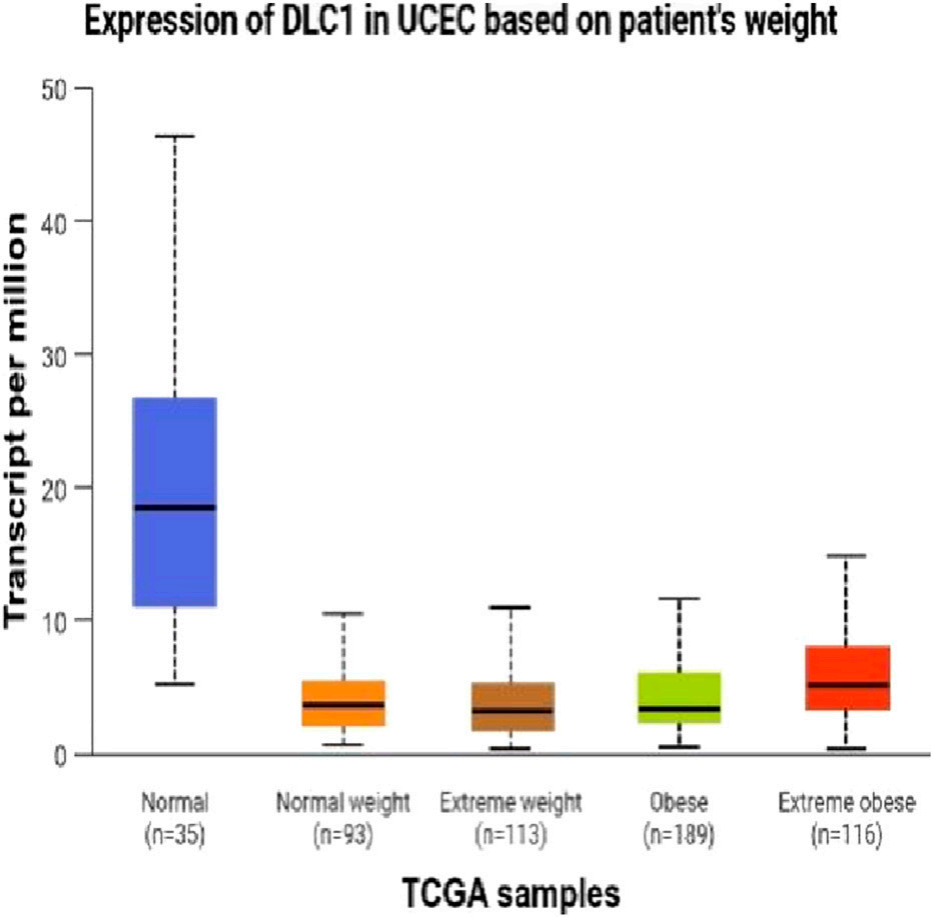
Expression of DLC1 in UCEC on patient’s weught.

**FIGURE 8:**
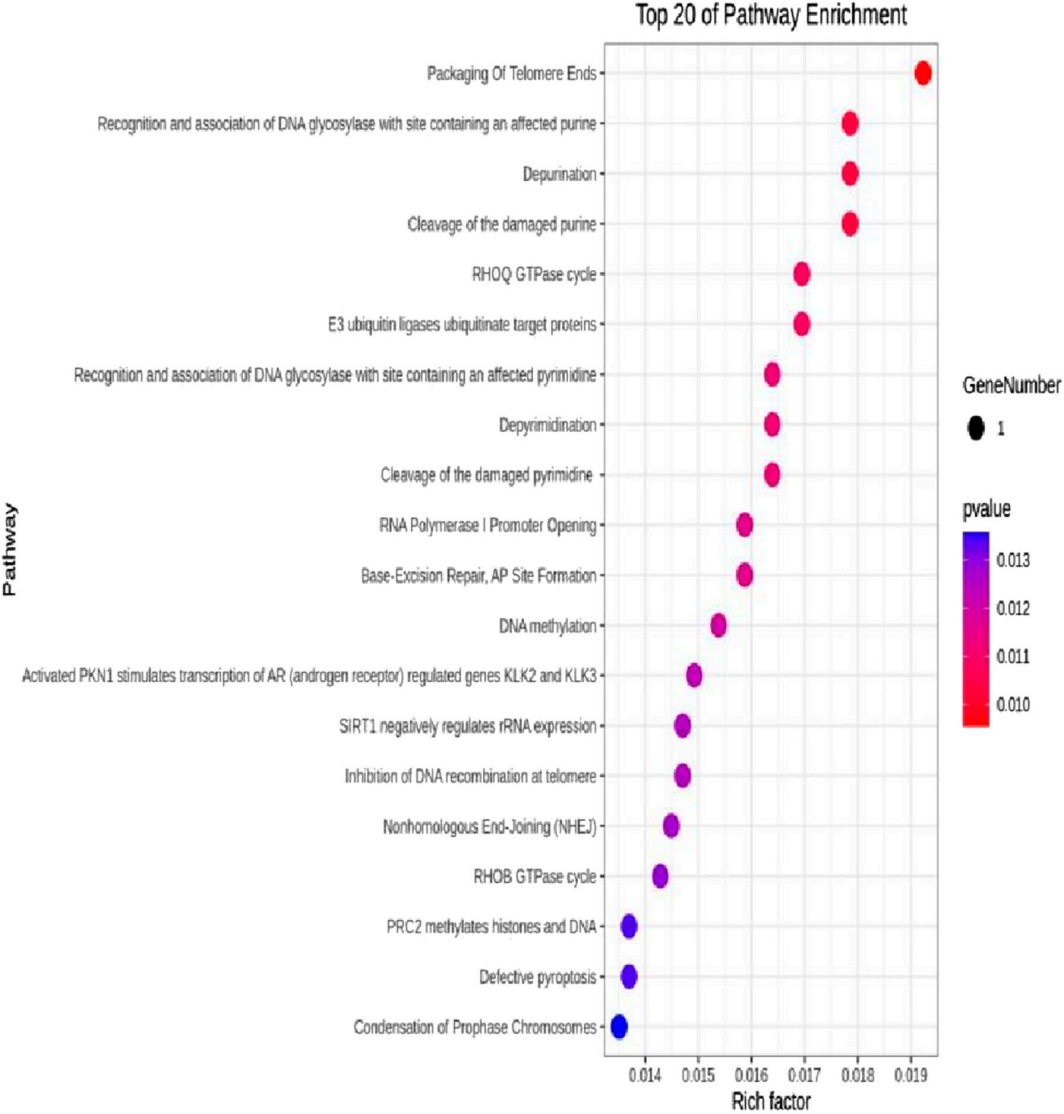
Top 20 Reactome enrichment pathways.

The DEseq2/edgeR software was utilized to analyze the differentially expressed genes between the experimental groups and the control group, including the 3D ADSCs-Exos + JEC group, 2D ADSCs-Exos + JEC group, and JEC control group. Under default conditions, genes with P < 0.05 and |log2 (fold change)| > 1 were defined as significantly differentially expressed. Specifically, genes with log2 (fold change) > 1 were labeled as upregulated (Up), while those with log2 (fold change) < −1 were labeled as downregulated (Down). Genes that did not meet these criteria were considered as non-significantly differentially expressed. The volcano plot presented in Figure 4 depicts the upregulation and downregulation of the differentially expressed genes. The levels of both NSUN5P2 and DLC1 in the tissues of 546 endometrial cancer patients showed significant differences compared to those in the normal control group (Figures 5–7). REACTOME is an open-source, freely accessible, manually curated, and peer-reviewed pathway database. Its goal is to provide visual and interpretable analyses for various pathways to support basic research, clinical studies, genomic analysis, modeling, systems biology, and other related fields. We conducted Reactome enrichment analysis using the ReactomePA package, and the results are presented in Figure 8. The significance of various pathways in Reactome lies in their roles in cell cycle regulation, DNA repair, apoptosis, and developmental processes.

## 4 Discussion

Exosomes are currently recognized as multipotent and abundant tools in the field of cell therapy and regenerative medicine. ADSCs can produce and secrete numerous exosomes, inheriting multiple functions of the cells (Hong et al., 2019). These exosomes, can secret various cytokines, maintain homeostasis, regulate immune responses and inflammation (Han et al., 2019; Blázquez et al., 2014; Wu et al., 2019; Zhao et al., 2018; Zhou Z. et al., 2021), promote neurogenesis and angiogenesis (Chen et al., 2019; Li, 2022), and are associated with cancer cell proliferation, invasion, and apoptosis (Hong et al., 2019). When compared to ADSCs, ADSCs-Exos exhibit higher stability and are easier to store, with no immune rejection, and their dose can be conveniently controlled. As a cell-free therapeutic approach, ADSCs-Exos hold notable advantages in terms of safety, efficacy, and application methods. Nonetheless, the low and inconsistent yield of ADSCs-Exos remains a significant challenge for their large-scale clinical application.

Recent evidence suggests that ADSCs-Exos can be internalized by endometrial stromal cells and epithelial cells, enhancing stromal cell proliferation while suppressing apoptosis. This process may contribute to endometrial regeneration and attenuation of fibrosis (Wang et al., 2023; Shao et al., 2021; Lin et al., 2021). However, current research on ADSCs-Exos in endometrial malignancies remains limited. Endometrial carcinoma, ranking among the top three prevalent gynecological malignancies,is classified into two categories. Type I, characterized by an estrogen-dependent nature, predominantly comprises endometrioid adenocarcinomas, with a smaller proportion of mucinous adenocarcinomas. Type II, non-estrogen-dependent, includes serous carcinoma, mucinous carcinoma, clear cell carcinoma, and carcinosarcoma. Presently, it is widely acknowledged that the onset of Type I endometrial cancer is closely tied to prolonged, uninterrupted exposure to estrogen, coupled with inadequate progesterone counteraction. Excess estrogen, stemming from various sources, is deemed the chief instigator of endometrial cancer. The etiology of Type II endometrial cancer, however, remains perplexing. Patients with endometrial cancer commonly exhibit coexisting conditions such as obesity, diabetes, and hypertension; these three disorders are therefore collectively known as the “endometrial cancer triad” (National Health Commission of the People’s Republic of China, 2020). A singular study explored the impact of ADSCs-Exos on human endometrial carcinoma cells, revealing that ADSCs-EXOs enhance the proliferation and suppress apoptosis of HEC-251 via the TGF-β/Smad signaling pathway (Wang et al., 2019). Notably, previous EC studies predominantly utilized ISK, HEC1-A, or other cell lines. The JEC cell line, derived from moderately differentiated human endometrial adenocarcinoma, exhibits unique estrogen receptor (ER)- and progesterone receptor (PR)-negative characteristics. In this study, we selected the JEC cell line to investigate the underlying mechanisms governing proliferation and metastasis in EC cells with varying differentiation statuses and ER/PR expression patterns.

In this experiment, we isolated and extracted extracellular vesicles (EVs), specifically ADSCs-Exos, from adipose-derived mesenchymal stem cells (ADSCs) cultured in both two-dimensional (2D) and three-dimensional (3D) environments. Ultracentrifugation effectively extracted EVsfrom both 2D and 3D ADSCs, demonstrating a significantly higher yield from 3D-cultured ADSCs, which suggests that 3D-cultured ADSCs are advantageous for large-scale EV extraction. Morphological examination is essential for identifying EVs. The extracted ADSCs-Exos were initially visualized under transmission electron microscopy, revealing their characteristic round or oval morphology with a lipid bilayer membrane, approximately 100 nm in diameter, consistent with the established morphological description of EVs in the literature. Nanoparticle tracking analysis (NTA) technology enabled real-time observation and imaging of EVs within a specific diameter range in suspension, allowing us to scan individual EV particles to determine their concentration and generate a size distribution chart. Using NTA, we characterized ADSCs-Exos derived from both 2D and 3D cultures, with results indicating a median diameter of 125.6 nm. The original particle concentrations were 1.1 × 10^11^ particles/mL in the 2D EV suspension and 3.1 × 10^11^ particles/mL in the 3D EV suspension. Western blot (WB) analyses confirmed that specific membrane proteins CD63 and CD81, along with the membrane-associated complex TSG101, were positively expressed in both 2D and 3D EVs, verifying that the extracted and separated substances were indeed ADSCs-Exos, and both 2D and 3D ADSCs-Exos could be rapidly internalized by JEC cells.

In this investigation,we demonstrate that both two-dimensional (2D) and three-dimensional (3D) adipose-derived stem cell extracellular vesicles (ADSCs-Exos) are readily internalized by endometrial adenocarcinoma cells (JEC). Regarding yield, 3D ADSCs-Exos exhibit superior performance compared to 2D ADSCs-Exos. Functionally, both 2D and 3D ADSCs-Exos significantly enhance the proliferation, invasion, and migration of JEC cells when compared to untreated controls. RNA sequencing indicates that NSUN5P2, Linc01694, and DLC1 in differential genes are associated with the cell cycle and the enhancement or inhibition of tumor cell proliferation and invasion (Gu et al., 2018; Liu et al., 2020; Wu et al., 2022), respectively. Upon searching the ULCAN website, we further discovered that the levels of both NSUN5P2 and DLC1 in the tissues of 546 endometrial cancer patients showed significant differences compared to those in the normal control group. Additionally, there was a certain correlation between DLC1 levels and the patients’ body weight. Reactome pathway analysis further reveals their relevance to the cell cycle, DNA repair, apoptosis, and other processes. We consider these findings as potential reasons for ADSCs-Exos to enhance the proliferation, migration, and invasion capabilities of endometrial cancer JEC cells. However, further investigation is necessary to elucidate the specific molecules and mechanisms involved.

Moreover, the efficacy of 3D ADSCs-Exos surpasses that of their 2D counterparts, aligning with previously reported outcomes. The 3D culture system mimics a more authentic cellular microenvironment *in vivo* through cell-to-cell communication and interaction, distinguishing it from the 2D system. Protein expression profiles also exhibit significant differences between 3D and 2D exosomes (Chen et al., 2021), which underscores the impact of exosomes on organismal metabolism through the expression of their internal proteins—a fundamental principle guiding the clinical application of exosomes. Consequently, 3D exosomes display superior effects, including enhanced anti-inflammatory properties, angiogenesis, and promotion of cell proliferation (Lin et al., 2024). We hypothesize that this may explain why 3D ADSCs-Exos exert a more potent influence on the proliferation, migration, and invasion of endometrial cancer cells compared to 2D ADSCs-Exos.

This study establishes a foundation for elucidating the role of ADSCs-Exos in endometrial adenocarcinoma cell regeneration *in vitro*, while simultaneously alerting us to their potential tumor-promoting risks in clinical applications. Future investigations should focus on functional validation and *in vivo* studies to elucidate the precise mechanisms by which ADSCs-EXOs influence endometrial carcinogenesis and progression. Moreover, given the hormone-sensitive nature of endometrial cancer, subsequent research must incorporate hormonal conditions—particularly estrogen exposure—to better recapitulate the clinical tumor microenvironment.

## 5 Conclusion

In summary, both two-dimensional (2D) and three-dimensional (3D) adipose-derived stem cell-derived exosomes (ADSCs-EXOs) exhibit regenerative effects on endometrial adenocarcinoma cells (JEC), significantly enhancing proliferation, invasion, and migration. These effects may be associated with altered expression levels of NSUN5P2 and DLC1. While ADSCs-Exos hold therapeutic potential, their clinical application warrants caution due to possible pro-tumorigenic risks. Further investigations are required to elucidate the precise mechanisms underlying their role in cancer initiation and progression.

## Data Availability

The raw RNA-seq data generated in this study have been publicly deposited and are accessible via the NCBI BioProject database under the accession number PRJNA1288755 (https://www.ncbi.nlm.nih.gov/bioproject/1288755). All other primary raw datasets supporting the findings of this work are included in the main text or supplementary materials of this article. For any additional inquiries regarding data availability or access, please direct correspondence to the corresponding author.
