# Supplementary figures and images for: Biological effects of exosomes derived from 2D and 3D culture adipose stem cells on JEC Cell proliferation and migration
Source: Front Bioeng Biotechnol. 2025 Jul 10;13:1541150. doi: 10.3389/fbioe.2025.1541150 (PMC12287758; doi:10.3389/fbioe.2025.1541150)

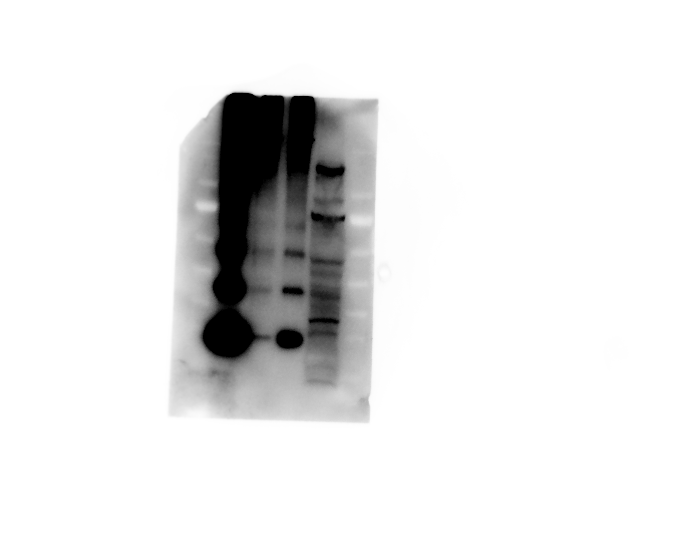

Supplement: Supplementary file 1 [file Image6.tif]

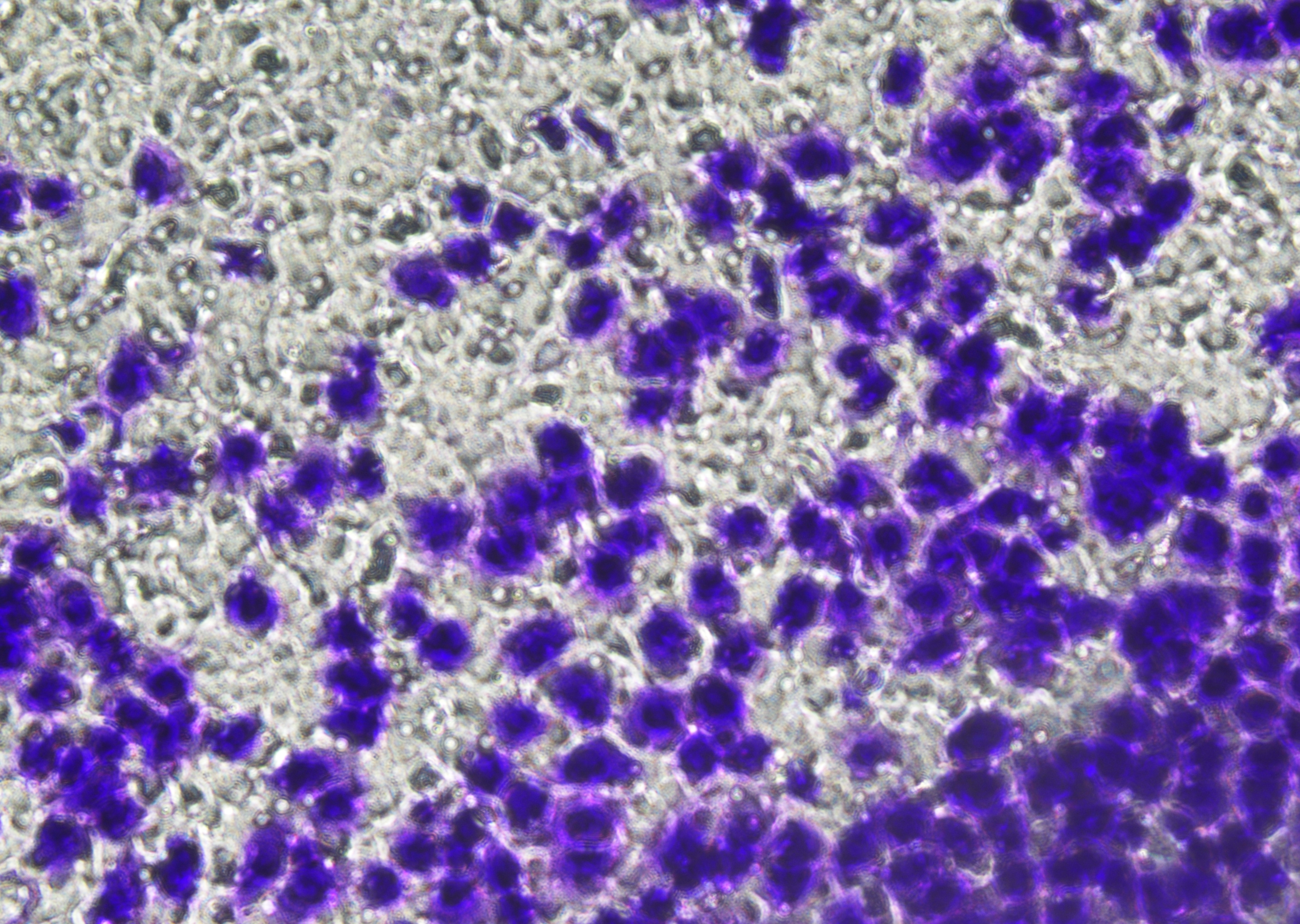

Supplement: Supplementary file 2 [file Image9.jpeg]

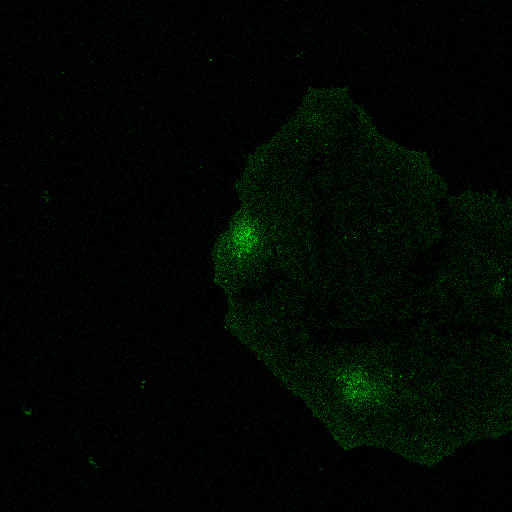

Supplement: Supplementary file 3 [file Image14.tif]

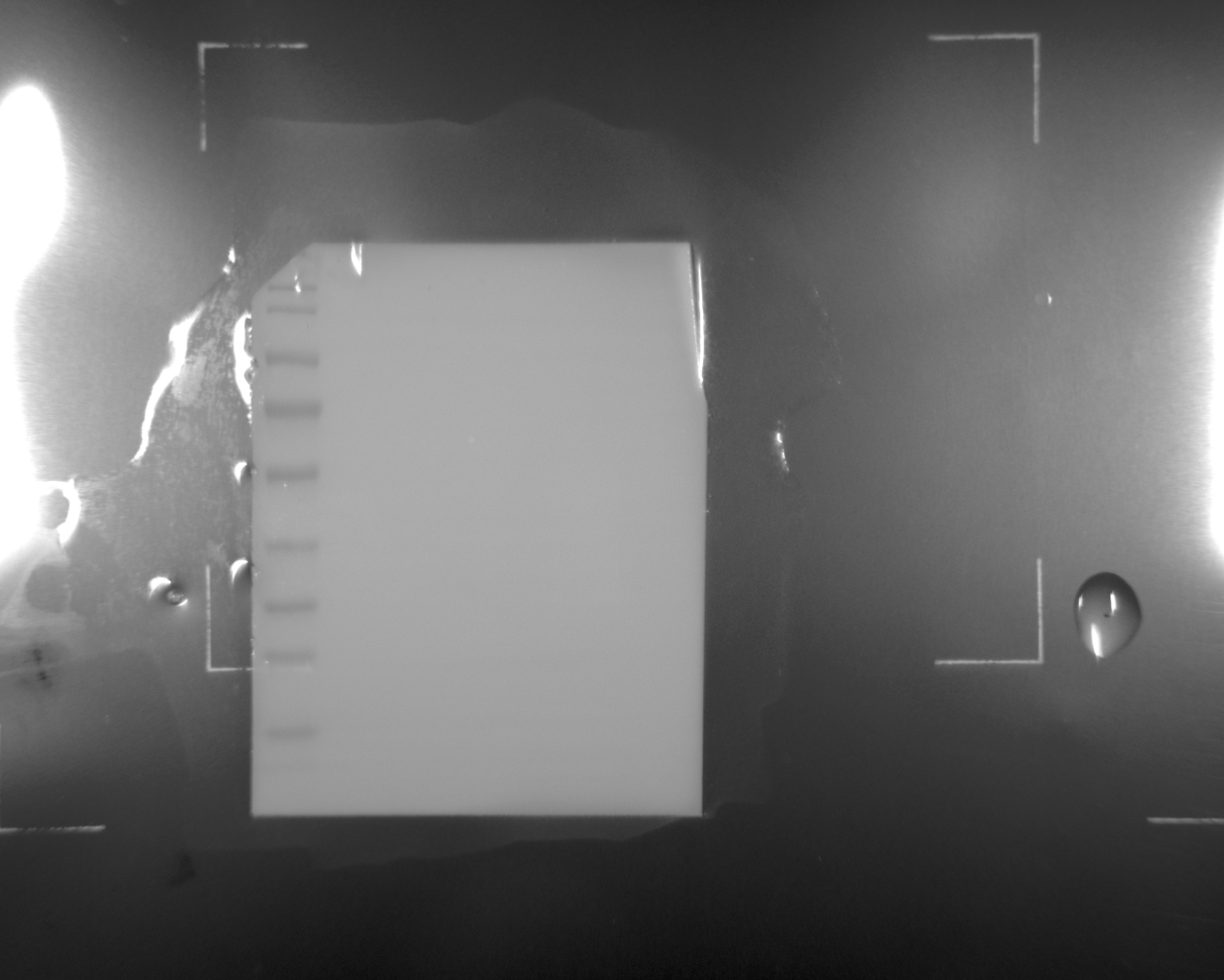

Supplement: Supplementary file 4 [file Image3.tif]

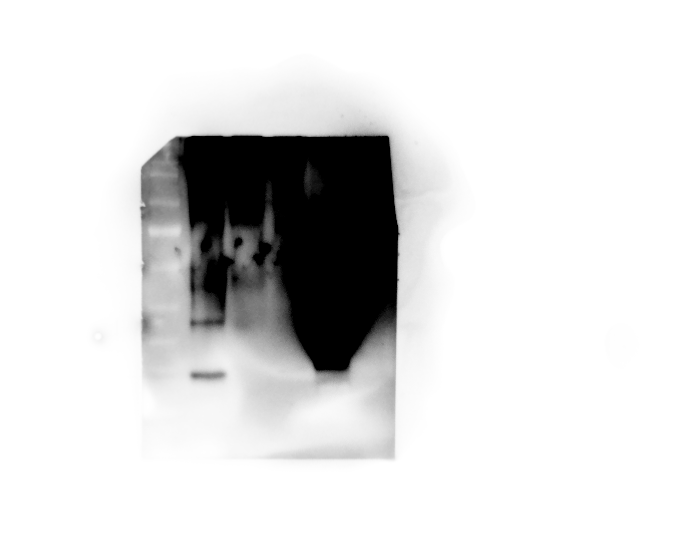

Supplement: Supplementary file 5 [file Image4.tif]

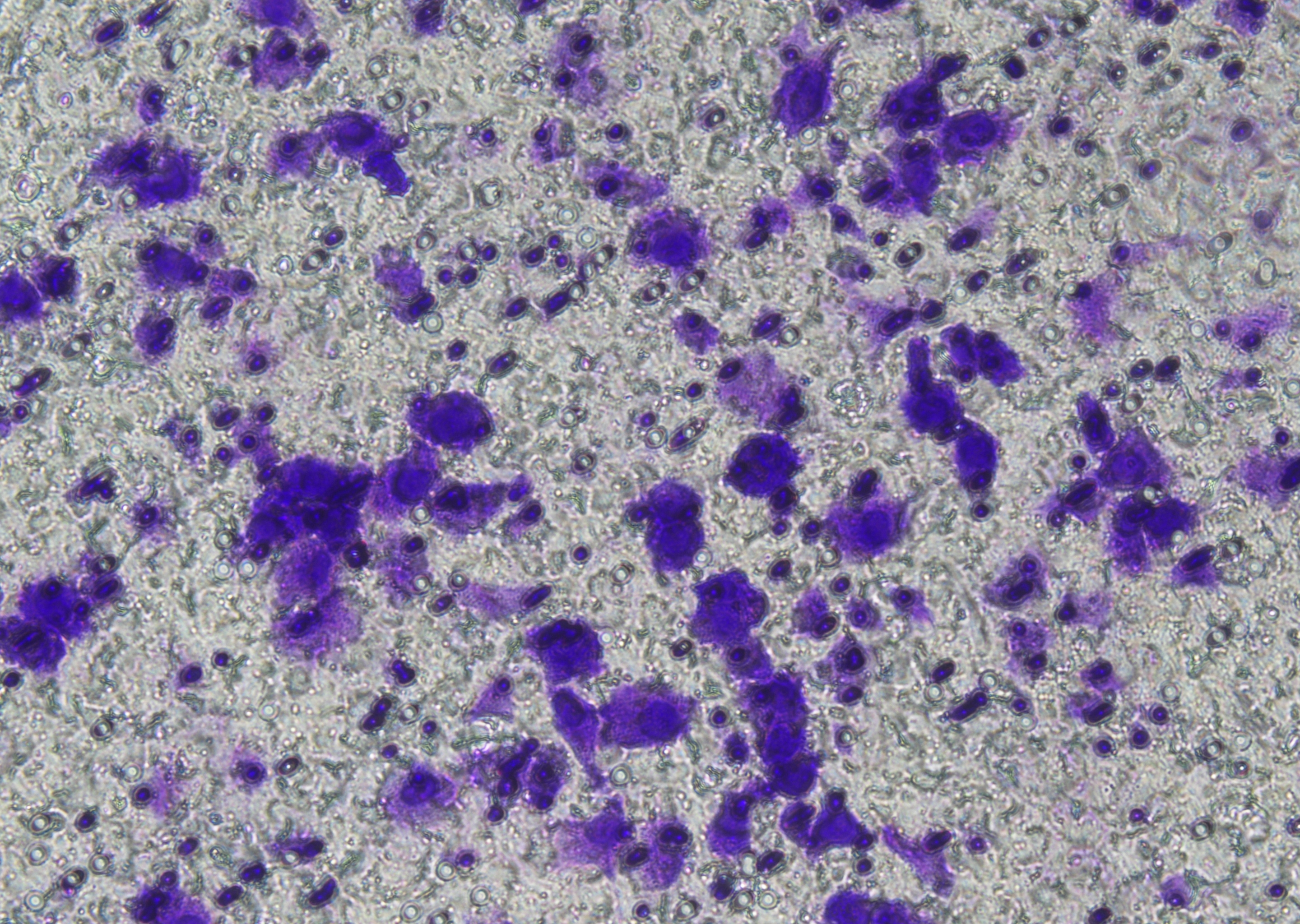

Supplement: Supplementary file 6 [file Image10.jpeg]

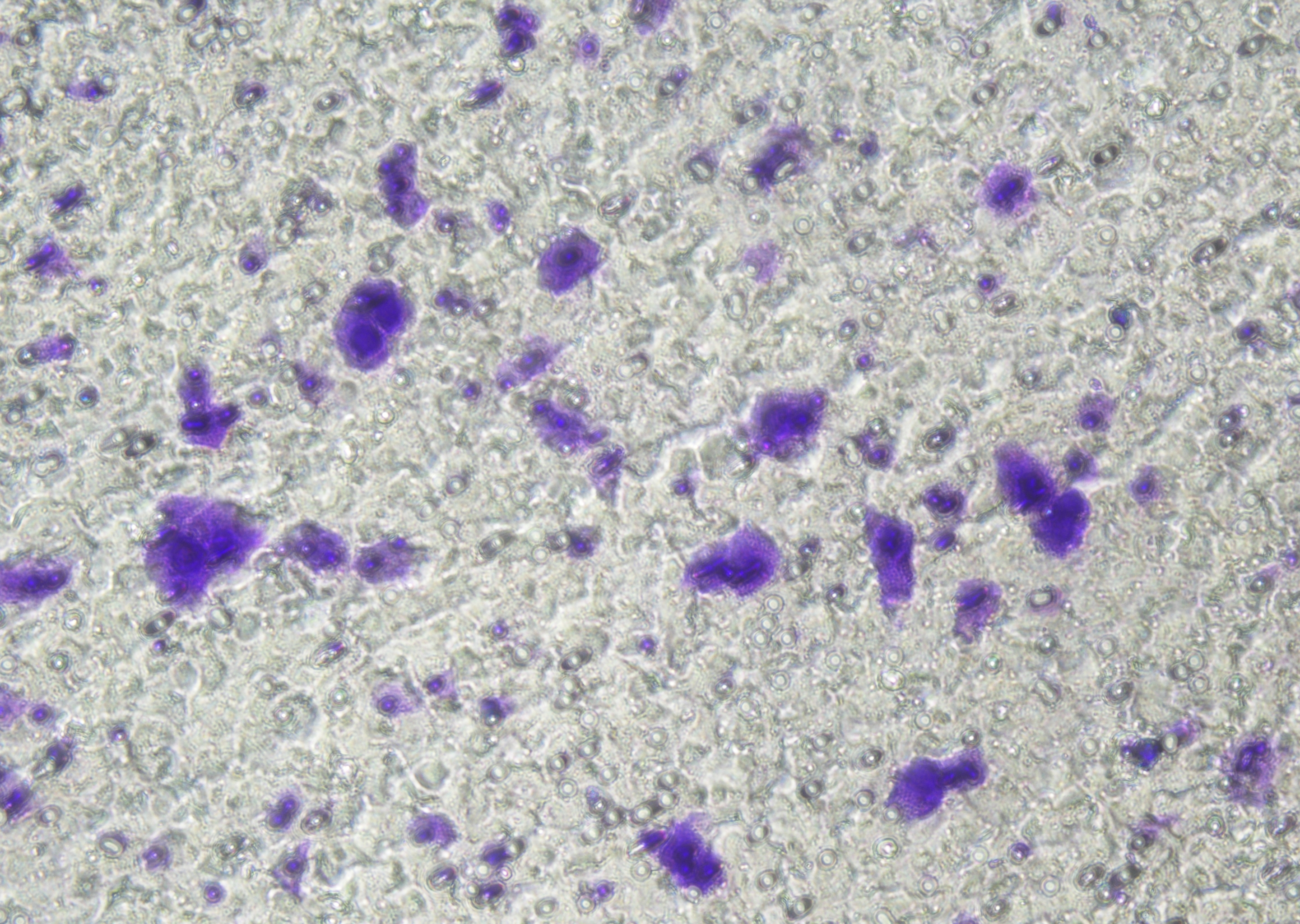

Supplement: Supplementary file 7 [file Image12.jpeg]

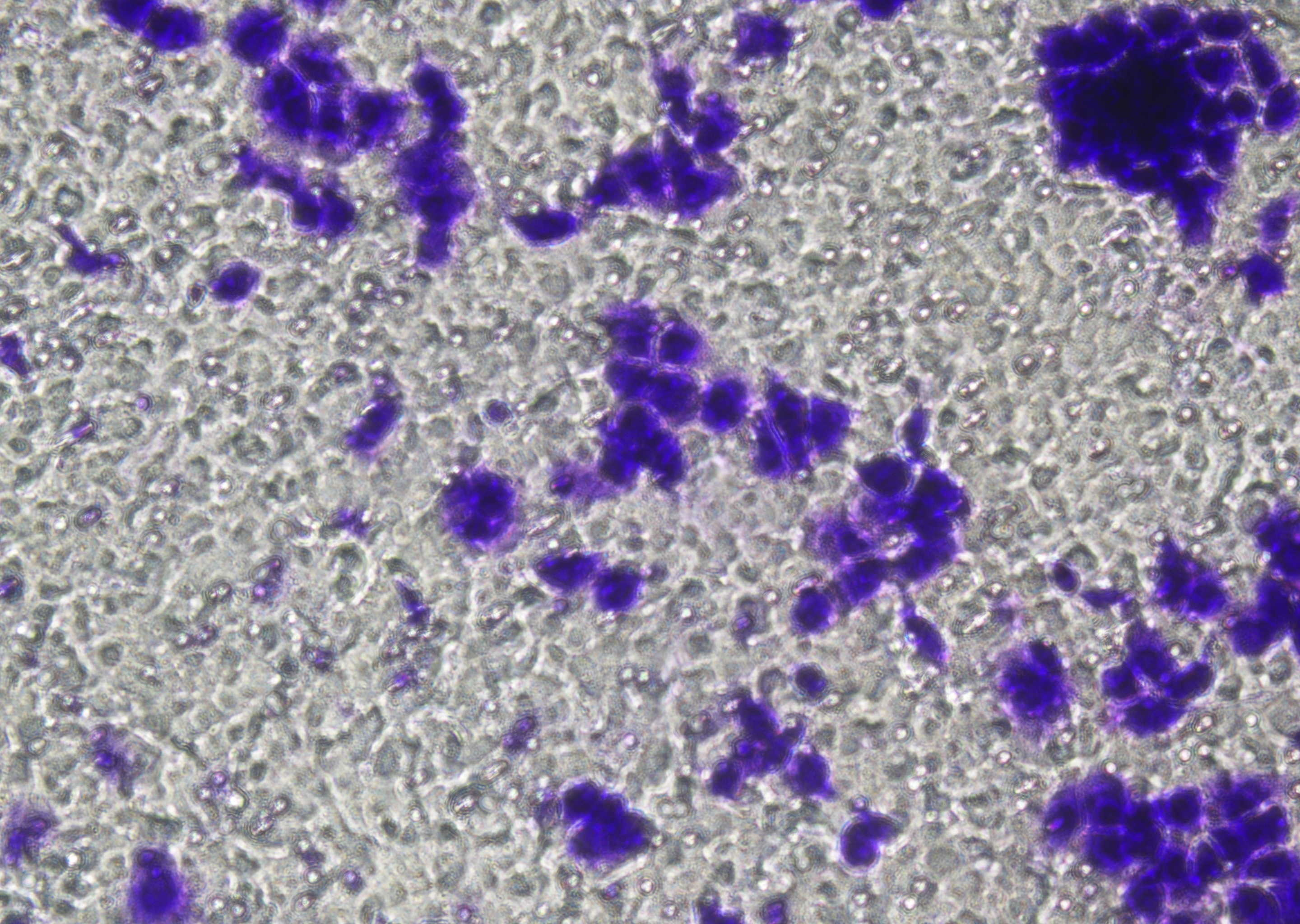

Supplement: Supplementary file 8 [file Image11.jpeg]

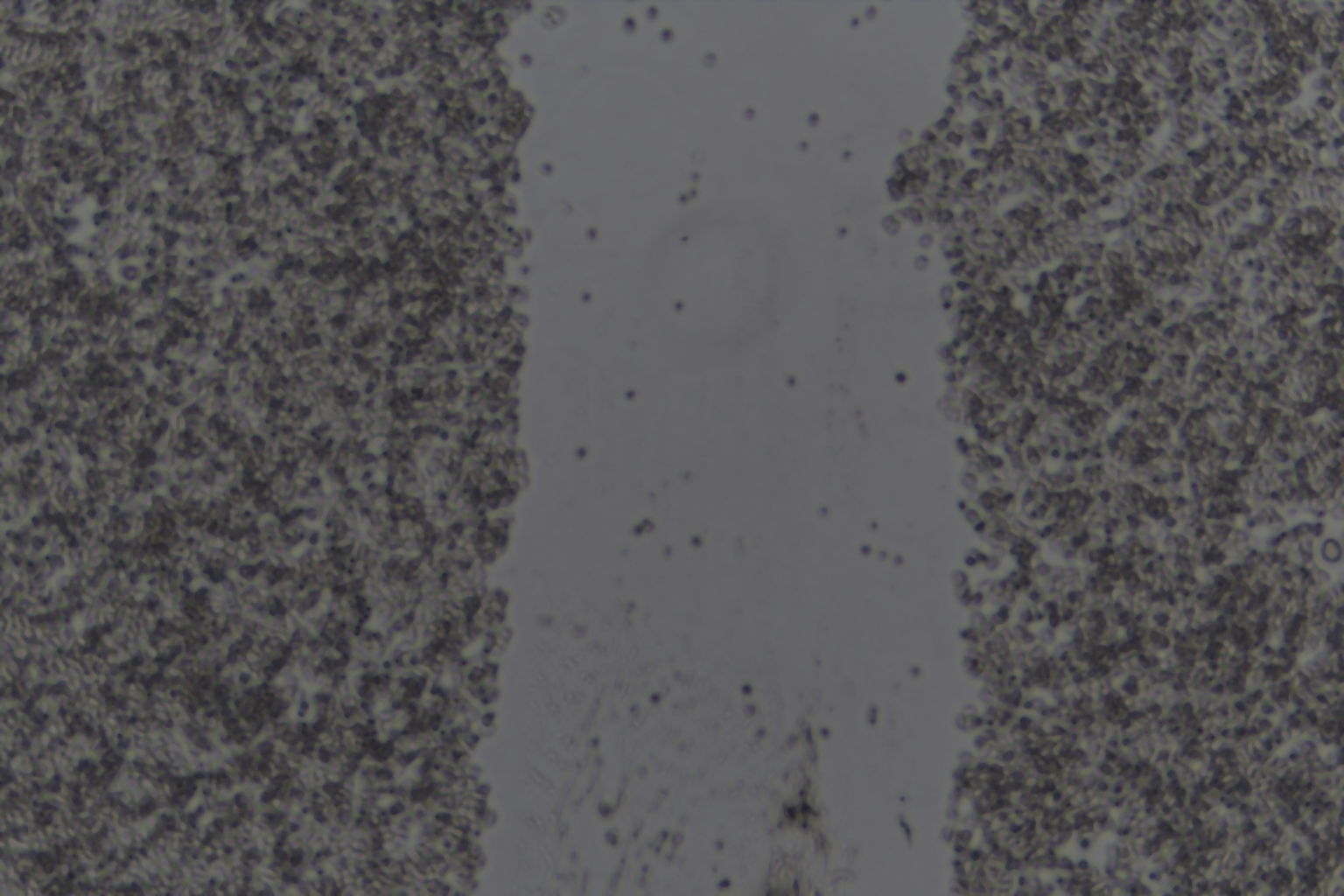

Supplement: Supplementary file 9 [file Image7.tif]

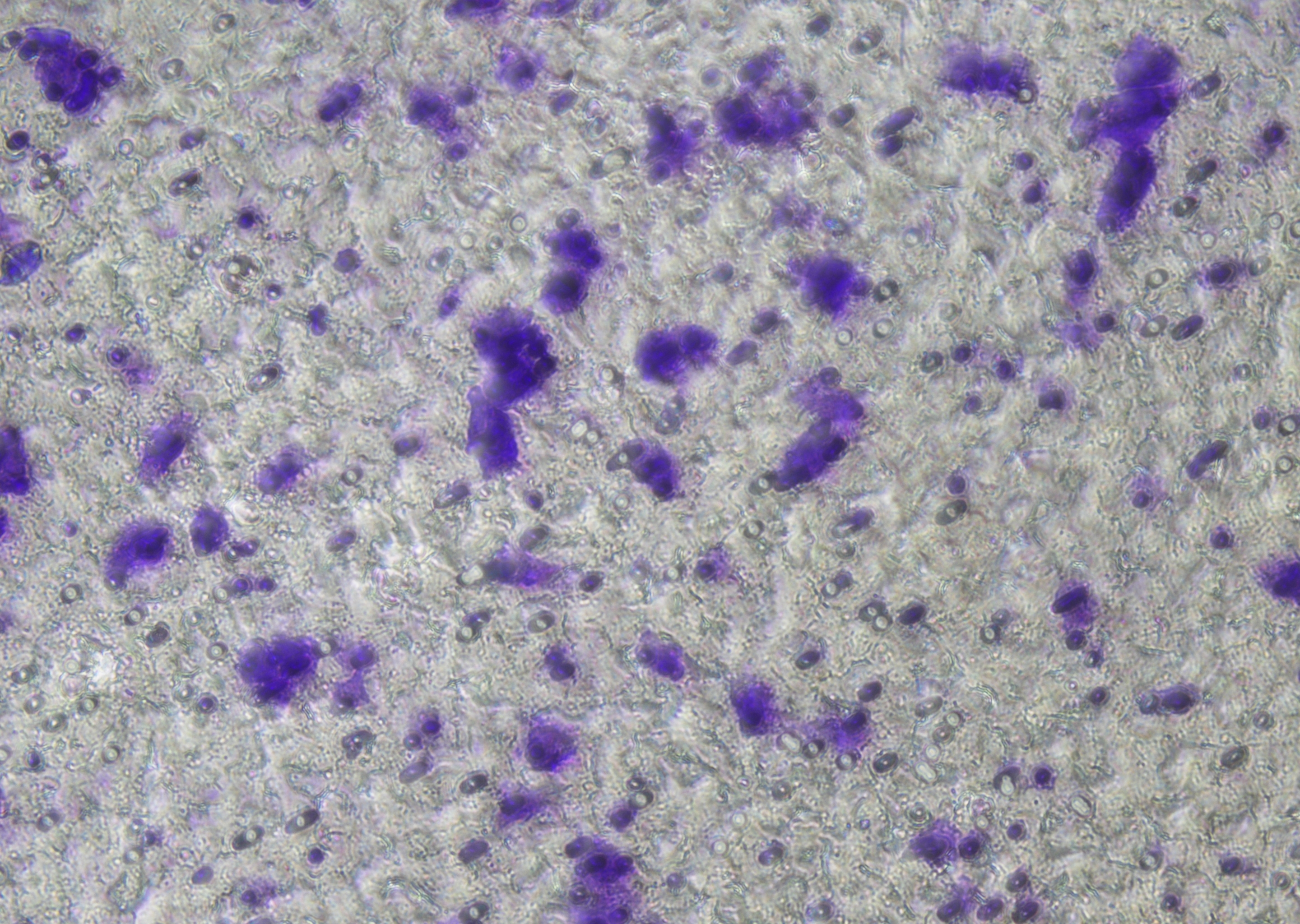

Supplement: Supplementary file 10 [file Image13.jpeg]

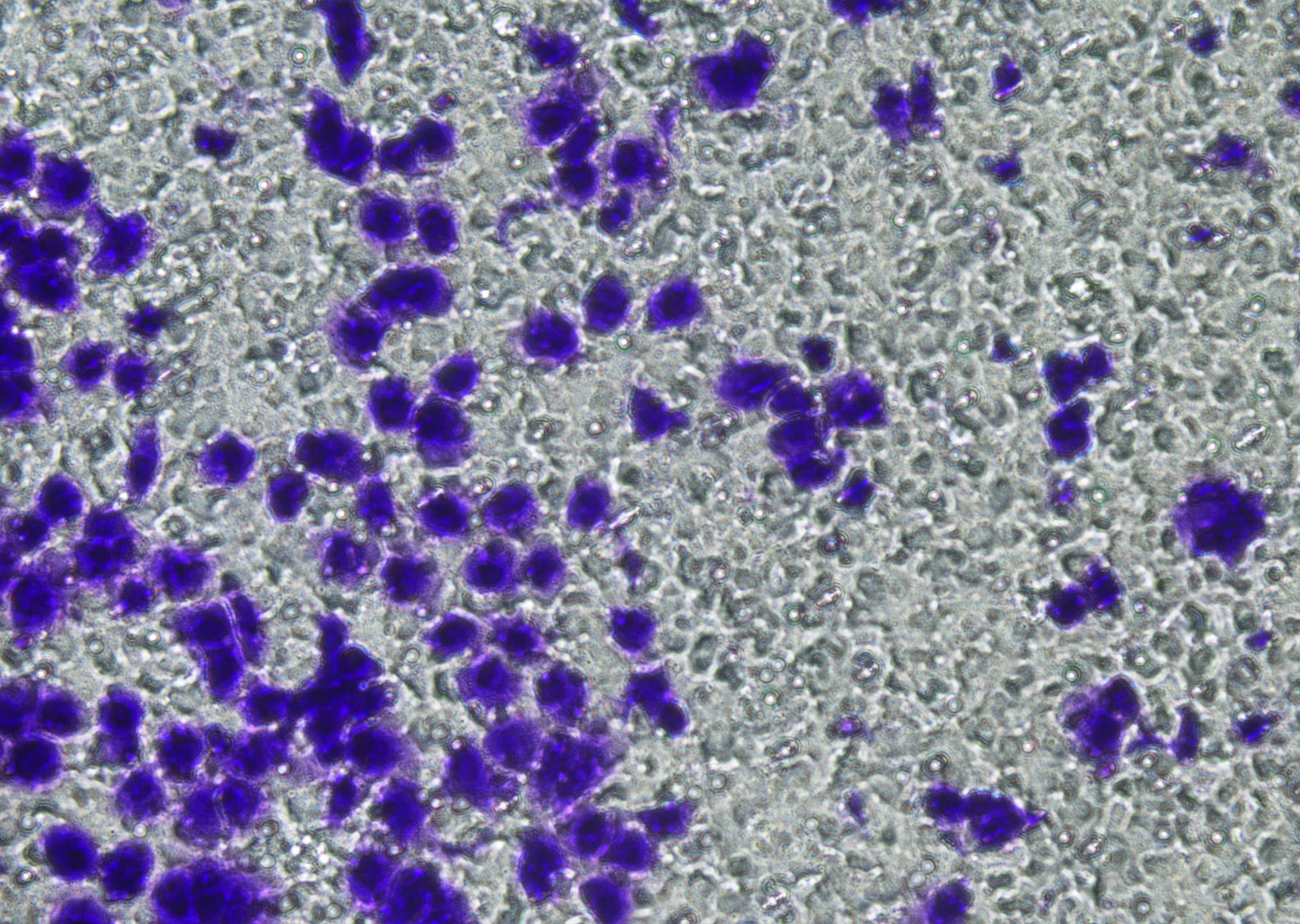

Supplement: Supplementary file 11 [file Image8.jpeg]

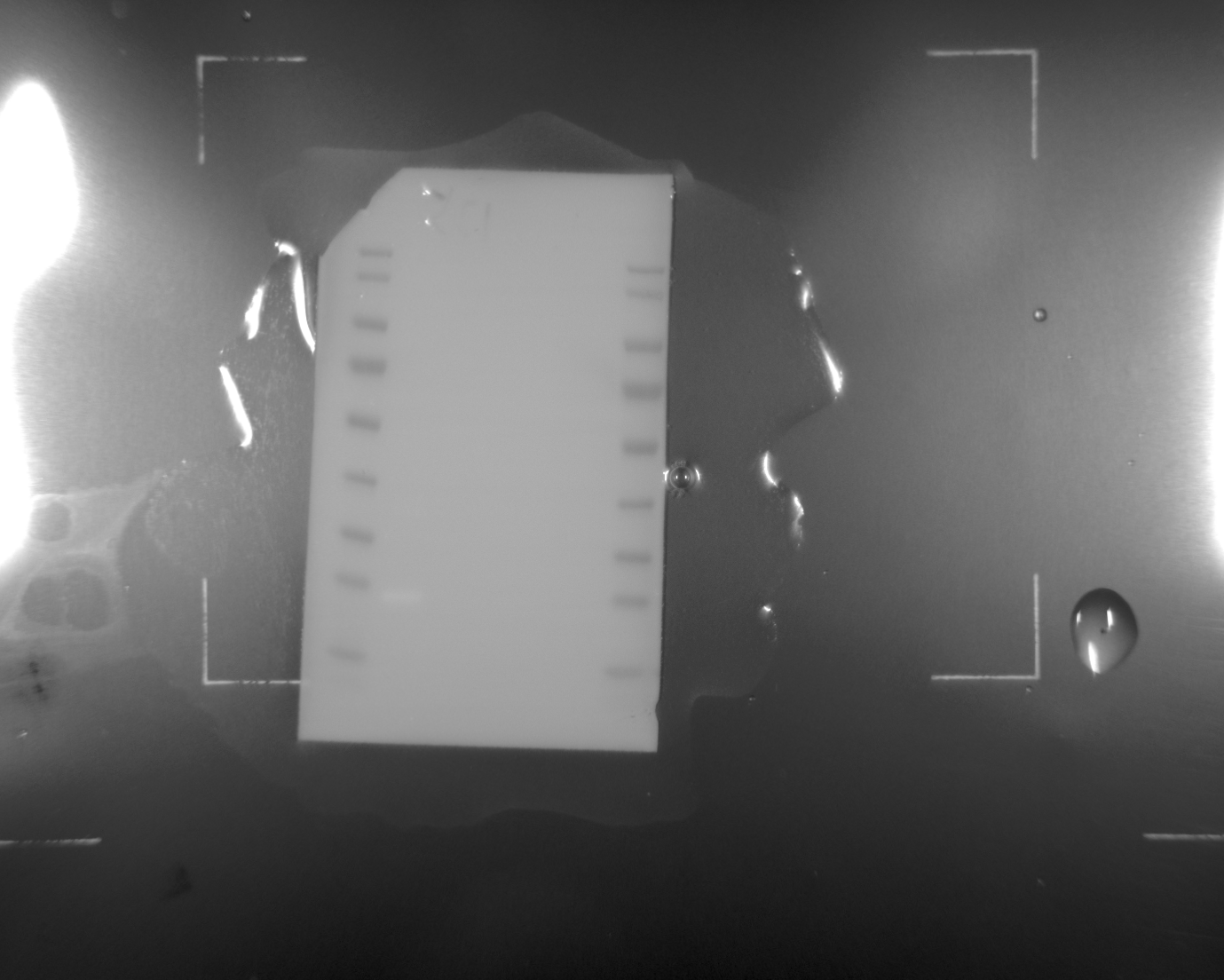

Supplement: Supplementary file 12 [file Image5.tif]

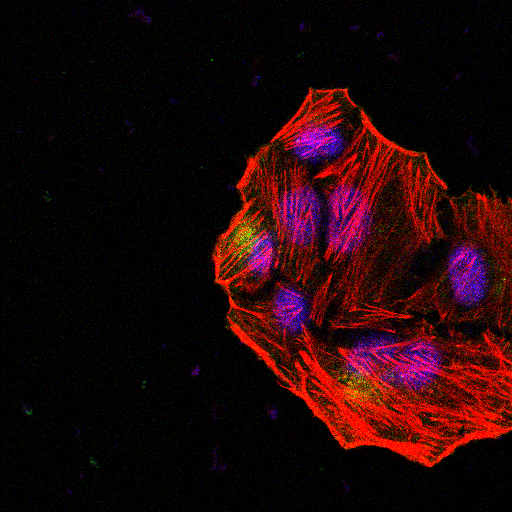

Supplement: Supplementary file 13 [file Image15.tif]
